# Multi-Platform Classification of IDH-Wild-Type Glioblastoma Based on ERK/MAPK Pathway: Diagnostic, Prognostic and Therapeutic Implications

**DOI:** 10.3390/cancers13184532

**Published:** 2021-09-09

**Authors:** Maria-Magdalena Georgescu

**Affiliations:** NeuroMarkers PLLC, Houston, TX 77025, USA; mmgeorgescu@yahoo.com; Tel.: +1-281-433-0492

**Keywords:** glioblastoma molecular classification, ERK/MAPK pathway, PI3K/PTEN pathway, receptor tyrosine kinase, EGFR, PDGFRA, FGFR3, MET, EPHB2, NF1

## Abstract

**Simple Summary:**

This study presents a unified classification of glioblastoma that streams multi-platform data from genomic, transcriptomic, histologic, and demographic analyses into targetable glioblastoma subgroups connected by signaling through canonical growth pathways. This structured and clear analysis addresses the current needs of neuro-oncological practice and offers practical guidelines for the diagnosis, prognosis, and therapeutic targeting of glioblastoma, being thus of great benefit to both patients and brain tumor practitioners alike.

**Abstract:**

Glioblastoma is the most aggressive and frequent glioma in the adult population. Because current therapy regimens confer only minimal survival benefit, molecular subgrouping to stratify patient prognosis and therapy design is warranted. This study presents a multi-platform classification of glioblastoma by analyzing a large, ethnicity-inclusive 101-adult-patient cohort. It defines seven non-redundant IDH-wild-type glioblastoma molecular subgroups, G1–G7, corresponding to the upstream receptor tyrosine kinase (RTK) and RAS-RAF segment of the ERK/MAPK signal transduction pathway. These glioblastoma molecular subgroups are classified as G1/EGFR, G2/FGFR3, G3/NF1, G4/RAF, G5/PDGFRA, G6/Multi-RTK, and G7/Other. The comprehensive genomic analysis was refined by expression landscaping of all RTK genes, as well as of the major associated growth pathway mediators, and used to hierarchically cluster the subgroups. Parallel demographic, clinical, and histologic pattern analyses were merged with the molecular subgrouping to yield the first inclusive multi-platform classification for IDH-wild-type glioblastoma. This straightforward classification with diagnostic and prognostic significance may be readily used in neuro-oncological practice and lays the foundation for personalized targeted therapy approaches.

## 1. Introduction

Glioblastoma is the most frequent malignant primary brain neoplasm in adults, with an incidence of 3–4 cases per 100,000 population, and 41% survival at 1 year [1]. The 2016 World Health Organization (WHO) Classification of Tumors of the Central Nervous System recognizes IDH-wild-type and IDH-mutant glioblastomas as separate molecular entities, with significant survival differences [2]. IDH-wild-type glioblastomas comprise over 90% of glioblastomas and show *TERT* promoter mutations, *CDKN2A/B* homozygous loss, *EGFR* amplification, *TP53* and *PTEN* mutations, as most frequent common and mostly concurrent alterations [2]. These mutations address basic cancer cell maintenance requirements: telomere extension by *TERT* overexpression, cell cycle progression by *CDKN2A/B* cell cycle-dependent kinase (CDK) 4/6 inhibitors loss and *TP53* alterations, the latter being also involved in gatekeeping the DNA-damage response (DDR), and cell survival by inactivation of PTEN, the main inhibitor of the phosphatidyl inositol 3-OH kinase (PI3K) proliferative and anti-apoptotic pathway [3,4]. EGFR is the most frequently altered receptor tyrosine kinase (RTK) in glioblastoma, but amplification and activating mutation or fusions have been also reported in other RTKs, such as PDGFRA, MET, FGFR, and NTRK1 [2].

The RTKs are categorized into 19 well-defined classes based on sequence and structural similarity of their ligand-binding extracellular domains [5]. The intracellular domains contain the highly homologous tyrosine kinase domain and more specific juxtamembrane and carboxyl (C)-terminal regions that contain tyrosine motifs. Upon ligand binding leading to RTK dimerization and activation, the phosphorylation of tyrosine motifs triggers the activation of downstream signaling pathways by docking SH2-domain-containing adaptor and enzymatic proteins. The extracellular signal-regulated kinase/mitogen-activated protein kinase (ERK/MAPK) and PI3K/AKT/mTOR are two parallel signaling pathways controlling proliferation, survival, metabolism, and invasion of cancer cells that are commonly activated by RTKs, whereas Src, STAT, and phospholipase C-γ pathways are additional pathways activated by most RTKs.

There are four conventional MAPK families: ERK1/2, p38, JUNK, and ERK5, all phosphorylated and activated by upstream kinases or MAP2Ks, in turn, phosphorylated and activated by a third layer of upstream MAP3Ks [6]. Within the ERK1/2 MAPK cascade, RTKs activate Ras, which, in its active GTP-bound form, binds and activates Raf kinases (MAP3K), which activate MEK1/2 (MAP2K), which further activate ERK1/2 (MAPK). Phosphorylated ERKs translocate to the nucleus to activate transcription factors or remain cytoplasmic to activate substrates involved in cell growth. The pathway is negatively controlled upstream by direct inhibition through NF1, a Ras GTPase-activating protein (GAP) but also by feedback loops resulting from ERK-dependent transcription of the ERK phosphatases DUSP4/5/6 and the Sprouty family members [7,8,9,10]. In contrast, the PI3K/AKT/mTOR pathway is a heterogeneous pathway involving both protein and lipid signal transduction mediators and is directly inhibited by the PTEN tumor suppressor, acting upstream as phosphoinositide phosphatase counteracting the effect of PI3K [3].

By using an integrated approach interrogating a controlled glioblastoma patient cohort, I propose a simplified multi-platform classification of glioblastoma tailored to map the ERK/MAPK pathway activation, with important implications for precision therapy.

## 2. Materials and Methods

Tumor specimens, histology and, immunohistochemistry (IHC): Surgical resection, biopsy or autopsy specimens were obtained from patients with glioblastoma, as previously described, in accordance with hospital regulations [7,11,12]. With one exception, the IDH-wild-type cases illustrated in this study correspond to first-diagnosis, untreated tumors. Formalin-fixed paraffin-embedded (FFPE) sections were stained with hematoxylin-eosin (H&E). Images were acquired with the Nikon Eclipse Ci microscope equipped with the Nikon Digital Sight DS-Fi2 camera (Nikon Instruments Inc., Melville, NY, USA), as previously described [13]. For histologic pattern analysis, digital images were acquired from all cases at various magnifications. Representative tumor fields were chosen, aiming at the viable tumor core, away from areas of necrosis and normal brain interface. The images were processed in batch for level adjustment and displayed stacked in an extended image library for comparison. For difficult patterns, both digitalized images and slides were cross-examined. IHC was performed on selected sections, as described [11,13]. The following primary antibodies were used: histone H3-K27M (Millipore/Sigma, Burlington, MA, USA), IDH1-R132H (DIA-H09, Dianova, Hamburg, Germany), p53 (DO-7), Ki-67 (30-9) (Roche/Ventana Medical Systems Inc., Tucson, AZ, USA), GFAP (EP672Y) (Ventana/Cell Marque, Rocklin, CA, USA).

Next-generation sequencing (NGS) and copy number (CN) variation: Nucleic acids were extracted from FFPE samples, as previously described [11]. Variant analysis and interpretation following NGS using the xT 596-gene or xE whole-exome panels (Tempus Labs, Chicago, IL, USA) or the customized 295-gene panel were performed as previously described [7,11,12]. CN analysis was performed as previously described [7,14]. Gene amplification was called for CN ≥ 7, and loss of heterozygosity (LOH) for alterations with loss of one allele. The tumor mutation burden is expressed as single-nucleotide protein-altering mutations per megabase DNA. The MGMT promoter methylation assay was performed by quantitative methylation-specific PCR using DNA extracted from FFPE samples (Integrated Oncology, Phoenix, AZ, USA).

Transcriptomics: Whole transcriptome RNA sequencing with RNA fusion detection was performed at Tempus Labs for all glioblastoma samples with more than 30% tumor on FFPE sections as described [7]. The expression was analyzed by a proprietary protocol. Briefly, the threshold for total RNA counts was set at ≥500 in at least one tumor sample, and pseudogenes and Y-chromosome genes were excluded. A ≥5-fold overexpression threshold was set for the average tumor values from subgroups relative to precursor low-grade control values, as previously described [7].

Statistical analysis: Differences between groups were assessed by using unpaired two-tailed *t*-test with or without Welch’s correction for variances significantly different, as described [15]. Multivariable correlation matrices and hierarchical clustering were generated for the glioblastoma subgroups by using the Pearson correlation coefficient. Kaplan–Meier survival analyses using the Log-rank (Mantel–Cox) test were performed as previously described [16,17]. Statistical significance was considered for *p* < 0.05. Confidence intervals for all tests were 95%. The graphic, statistic, and hierarchical clustering software included Microsoft Excel (Microsoft Corp., Redmond, WA, USA), GraphPad Prism (Version 8.3.0, GraphPad Software, La Jolla, CA, USA), and Instant Clue [18].

## 3. Results

### 3.1. Non-Redundant Molecular Classification of Glioblastoma Based on ERK/MAPK Pathway

**Figure 1:**
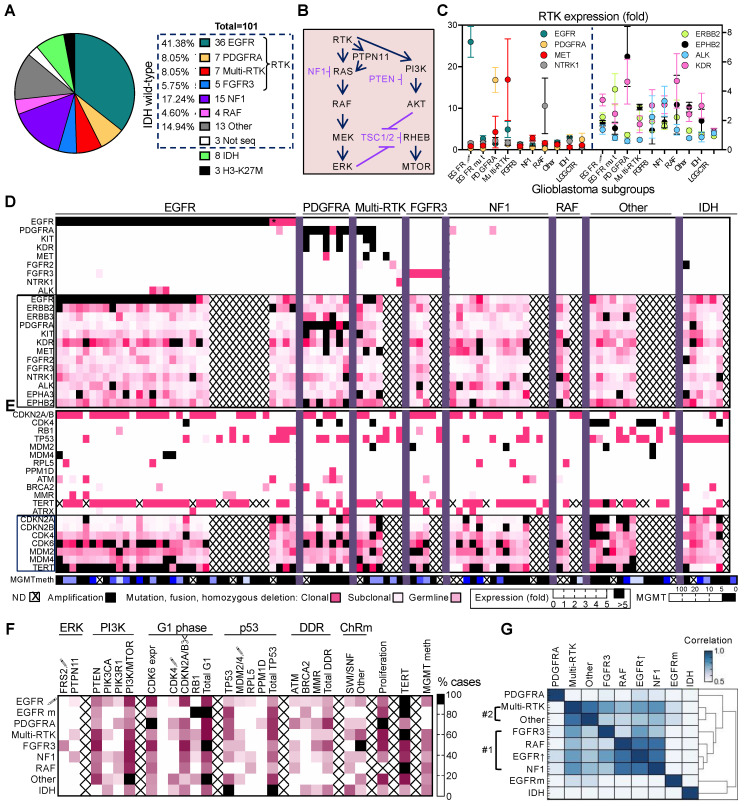
Glioblastoma molecular subgroups—molecular characteristics and pathway-based clustering. (**A**) Subgrouping of the glioblastoma cohort in non-overlapping molecular alterations corresponding to ERK/MAPK signaling pathway. (**B**) Diagram of the canonical ERK/MAPK and PI3K signaling pathways. Inhibitors are indicated in purple; arrows and blunt arrows represent activation or inhibition, respectively. (**C**) RTK fold-expression in glioblastoma subgroups, as mean ± SEM values, showing RTKs with high or intermediate overexpression on the left or right *y*-axis, respectively. EGFR↑, EGFR with gene amplification; EGFRm, EGFR with mutation only; LGG CTR, low-grade glioma expression control subgroup. (**D**,**E**) Heatmaps of subgrouped individual tumors showing mutation–expression correlations for RTKs (**D**) and cell cycle G1 phase, p53, DDR and telomere elongation pathways (**E**). Colored or black squares mark presence of genomic alterations in the upper part of the heatmaps. Asterisk (*) marks the multifocal EGFR#33 case with EGFR amplification in the main focus and EGFR mutation in the secondary focus, for which the expression profile is shown. Genes boxed in pink show expression data. MGMT promoter methylation (meth) is also shown, with positive values ≥ 5. ND, not determined. (**F**) Heatmap of % cases with indicated alterations grouped in pathways, based on the values from Table 1 for the glioblastoma subgroups. ↑, gene amplification (CN ≥ 7); ↓, homozygous loss; ChRm, chromatin remodeling. The gene composition of the DDR and ChRm pathways is described in Table 1. Mean CDK6 and proliferation markers expression values are also included. (**G**) Hierarchical clustering of glioblastoma subgroups by multivariable Pearson correlation analysis. Note two subgroup clusters #1 and #2, and individual segregation of the PDGFRA, EGFRm and IDH subgroups.

The tumors from a 101-adult-patient cohort with WHO grade IV diffuse glioma were initially classified based on IHC into IDH wild-type glioblastoma (90 cases), IDH-mutant glioblastoma (eight cases), and diffuse midline glioma (DMG) with histone H3 K27M mutation (three cases) (Figure 1A). NGS genomic results were obtained for 112 tumors from 97 patients and whole transcriptomics results were obtained for 82 tumors from 70 of these patients. The integrated genomic and transcriptomic analysis of the tumors showed *EGFR*, *PDGFRA*, *FGFR3*, *NF1*, and *BRAF/RAF1* mutually exclusive alterations within the glioblastoma IDH-wild-type category (Figure 1 and Table S1). An additional subgroup, Multi-RTK, showed alterations in multiple RTKs, including *MET*. The combined NF1, RAF, and RTK alterations from EGFR, PDGFRA, FGFR3, and Multi-RTK subgroups that activate the ERK/MAPK signaling pathway (Figure 1B) accounted for 85% of the glioblastoma IDH-wild-type cases. In the remaining cases, alterations targeting the ERK/MAPK pathway were not found, and this subset, labeled as Other, represented approximately 15% of IDH-wild-type glioblastoma cases (Figure 1A).

The RTK subgroups accounted for 63.2% of the glioblastoma IDH-wild-type cases, and the EGFR subgroup alone, for 41.4%, being thus the largest of all glioblastoma molecular subgroups (Figure 1A). The vast majority of the EGFR tumors from 91.7% of EGFR subgroup cases harbored *EGFR* amplification (EGFR↑), and only three cases showed *EGFR* gain-of-function mutations without amplification (EGFRm) (Table S1). Two tumors, EGFR#5 and EGFR#33, were multifocal with the main focus showing *EGFR* amplification and a secondary focus showing *EGFR* mutation without amplification. Interestingly, the majority of tumors with *EGFR* amplification also showed another *EGFR* genetic alteration, such as the splice variants vIII, vIVa, pathogenic mutations with or without amplification, C-terminal deletion, or *EGFR-SEPT14*, *EGFR-VOPP1*, *CTDSP2-EGFR*, and *SEC61G-EGFR* fusions. The next largest RTK subgroups are the PDGFRA and Multi-RTK, each accounting for 8% (Figure 1A). *PDGFRA* amplification with or without adjacent *KIT* and *KDR* amplification was the most frequent genetic alteration in the PDGFRA subgroup, and was found in 85.7% of the PDGFRA subgroup cases. As observed for *EGFR*, simultaneous *PDGFRA* amplification and missense mutations were frequently noted (Table S1). A common type of missense mutation targeted the di-sulfide bond cysteines of the extracellular domains of both EGFR and PDGFRα. The Multi-RTK subgroup is an eclectic group harboring various combinations of RTK alterations. It included three cases with *PDGFRA* amplification without overexpression. The smallest RTK subgroup is composed of cases with FGFR3 fusions or mutations (Table S1) and it has been characterized in detail elsewhere [7].

The NF1 and RAF subgroups accounted for almost one-quarter of the glioblastoma IDH-wild-type cases, with the NF1 subgroup being the second largest after the EGFR subgroup (Figure 1A). Two of the 15 NF1 cases were syndromic, and these patients presented other neurofibromatosis type 1 manifestations in addition to brain tumors. Almost all NF1 tumors had two *NF1* hits, either by different mutations or by LOH (Table S1). Twenty distinct *NF1* alterations were detected, most resulting in protein truncation. Among these, two *NF1* frameshift fusions were noted, underscoring the importance of fusion detection for correct classification in the NF1 subgroup. All four tumors in the RAF subgroup also showed distinct gain-of-function mutations, three in *BRAF*, and one in *RAF1*, the latter accompanied by gene amplification (Table S1).

The landscape of RTK expression in the glioblastoma subgroups was compiled by examining the relative expression levels of all the members from the 19 RTK classes (Table S2 and Figure S1). In the EGFR subgroup, *EGFR* high overexpression was associated with gene amplification in all but one case, with an average of 26 ± 3.7 and a range between 4.5- and 72-fold overexpression (Figure 1C,D). Conversely, low or no *EGFR* overexpression characterized the samples displaying activating *EGFR* gain-of-function mutations in the absence of amplification, indicating a strong correlation between *EGFR* amplification and overexpression in the EGFR subgroup. Moderate levels of EGFR overexpression were present in some cases of the Multi-RTK subgroup, and only in isolated cases in other subgroups, in the absence of gene amplification. In contrast to *EGFR*, only two-thirds of the cases with *PDGFRA* amplification showed overexpression, with an average of 16.8 ± 3 and a range between 4.9- and 25.7-fold (Figure 1C,D). The remaining one-third of cases were classified in the Multi-RTK subgroup, as they showed genetic alterations and/or overexpression of other major RTKs, most commonly *MET*, but also *EGFR*, *KIT*, *FGFR2*, *NTRK1*, *EPHA3*, and *EPHB2* (Figure 1C,D and Table S1). The *KIT* and *KDR* gene loci are contiguous with the *PDGFRA* locus, and their amplifications showed generally the same expression trend as *PDGFRA*, except for the Multi-RTK#1 case that showed high *KIT* overexpression in the absence of gene amplification. *MET* amplification was detected in two cases from the Multi-RTK subgroup and correlated with over 40-fold expression levels. Relatively high *MET* overexpression levels were also noted in four additional cases, ranging from 5.4- to 28-fold, without amplification but with low CN gain on chromosome 7. In the PDGFRA#4 case, *MET* overexpression was most likely caused by the presence of a *PTPRZ1-MET* fusion, as *PTPRZ1* is among the highest expressed genes in glioma, and the fusion places *MET* under the control of the *PTPRZ1* promoter (Table S1). *FGFR2* was the only other RTK with amplification and very high overexpression in the cohort, and the IDH#1 case harboring it has been previously described [7].

A number of RTKs showed >5-fold overexpression in some cases in the absence of gene amplification (Figure 1C,D and Figure S1). The other three members of the EGFR RTK class appeared upregulated differentially in the various glioblastoma subgroups, with *ERBB2* mild overexpression in the EGFR subgroup, especially associated with EGFRm cases, *ERBB3* mild overexpression in PDGFRA and Other subgroups, and *ERBB4*, in the IDH subgroup. *NTRK1* was upregulated in isolated cases in almost all glioblastoma subgroups but more prominently in the two cases with RNA expression data from the RAF subgroup. *NTRK1* genetic alterations were noted in only one case, the Multi-RTK#7 that showed *LMNA-NTRK1* fusion. Two of the ephrin class RTKs, *EPHA3*, and *EPHB2*, also showed overexpression in scattered cases across the glioblastoma subgroups but more prominently in the Multi-RTK and PDGFRA subgroups, respectively. Similarly, *ALK* showed overexpression in a few isolated cases and more clustered in the NF1 subgroup. Three EGFR cases also showed *ALK* variants of unknown significance or likely pathogenic (Table S1). Interestingly, *KDR*, besides the 10-fold overexpression in the cancer cells from the PDGFRA#4 and #6 cases with gene amplification, showed mild to moderate overexpression without gene amplification, with a 7.7-fold upper range, in the majority of cases from many subgroups. Most likely, this overexpression stems from the endothelial compartment, reflecting the active vascular proliferation program in these tumors. Interestingly, the pseudokinase RTKs PTK7, ROR1, and ROR2 that activate the Wnt pathway rather than the canonical MAPK and PI3K pathways [19] were mildly to moderately overexpressed in all glioblastoma subgroups (Figure S1).

### 3.2. Glioblastoma Subgroup Clustering Based on Pathway Analysis

The most frequent genetic alterations from glioblastoma were mapped to the different molecular subgroups (Table 1). *TERT* promoter mutations were the most frequent alteration, accounting for 81.2% of cases. *TERT* overexpression usually correlated with promoter mutations but also showed high values in a few cases without *TERT* promoter mutations (Figure 1E). *ATRX* mutations were rare and mostly complementary to *TERT* mutations. In general, *TERT* overexpression was the main mechanism of telomere elongation in IDH wild-type glioblastoma, except for the PDGFRA subgroup. The PI3K/mTOR canonical pathway showed genomic alterations in 76% of IDH-wild-type glioblastoma cases, mainly through *PTEN* mutations in 53% of cases (Table 1). The *PTEN* alterations peaked in the FGFR3, Multi-RTK, and Other subgroups. In contrast, *PIK3CA* mutations were rather clustered in the EGFR subgroup, and *PIK3R1* mutations, in the PDGFRA and NF1 subgroups.

Mutations in the cell cycle G1 phase genes *CDKN2A/2B*, *CDK4*, and *RB1* were mutually exclusive in this series, except for two heterozygous germline *RB1* point mutations, and were present in 79.3% of glioblastoma IDH-wild-type cases (Table 1 and Figure 1E). With one exception in the FGFR3 subgroup previously discussed [7], all *CDKN2A* homozygous losses were extended to the *CDKN2B* adjacent gene. Moreover, there was a perfect correlation between *CDKN2A/2B* homozygous loss and decreased RNA expression levels, although decreased levels were also seen in the absence of gene loss in two IDH-wild-type and one IDH-mutant glioblastoma cases. The type of mutated G1 phase gene was relatively specific in some subgroups. In particular, in the EGFR subgroup, EGFR↑ cases showed preferential *CDKN2A/2B* homozygous loss and EGFRm cases showed *RB1* inactivating mutations with LOH. The cases with *CDK4* amplification clustered in the Other subgroup that contained six of the ten total IDH-wild-type glioblastoma cases with *CDK4* amplification. This subgroup also presented cases with *RB1* mutation and only a minority with *CDKN2A/2B* loss. *CDK4* amplification correlated perfectly with overexpression. The other G1 phase kinase gene, *CDK6*, showed overexpression in the absence of genomic abnormalities especially in the PDGFRA, EGFR↑, and FGFR3 subgroups (Figure 1E,F).

The p53 cell cycle and cell proliferation gatekeeping pathway, defined here by mutations in *TP53* itself, as well as mutually exclusive mutations in *MDM2*, *MDM4*, *RPL5*, and *PPM1D*, was altered in approximately half of the IDH-wild-type and all IDH-mutant glioblastoma cases (Table 1 and Figure 1E). The *CDKN2A* gene locus also encodes p14ARF, a regulator of MDM2 that promotes its degradation and therefore stabilization of p53 [20]. With the exception of the NF1 and IDH subgroups, mutations in *TP53* tended to occur in the cases without *CDKN2A* loss, explaining the inverse relationship in mutation frequency between *TP53* and *CDKN2A* in most subgroups. Interestingly, *MDM4* mutations clustered in the EGFR subgroup and *MDM2* mutations were exclusively noted in the non-EGFR subgroups. Mutations in DDR genes *ATM* and *BRCA2* and in mismatch repair genes were clustered in the PDGFRA, IDH, and FGFR3 subgroups, respectively. In contrast, mutations of *STAG2*, encoding a subunit of the cohesin complex controlling sister chromatid separation during cell division, were scattered among almost all glioblastoma subgroups. Gene mutations in multiple chromatin remodeling mediators were present in all FGFR3 cases, and mutations especially in components of the SWI/SNF complex were noted in over half of IDH subgroup cases.

Overall, the tumor mutation burden of the subgroups was similar, with median values between 2.8 and 5.8 mutations/megabase DNA (Figure S2). Only three tumors representing 3.2% of the cohort had high tumor mutation burden values over 10 mutations/megabase DNA.

The relative specificity of mutation partition prompted the assembly of a correlation matrix for glioblastoma subgroup hierarchical clustering (Figure 1F). Besides gene mutations in the pathways discussed above, and *CDK6* average expression, two additional parameters were included, *MGMT* promoter methylation and proliferation. As compared to roughly half of the tumors in the Multi-RTK, FGFR3, RAF, but also EGFR↑ subgroups, none of the tumors from EGFRm or PDGFRA subgroups showed *MGMT* promoter methylation (Table 1 and Figure 1E). The extent of *MGMT* promoter methylation was also variable, with some tumors displaying only marginally positive values. The proliferation was assessed for the cases with expression data as a compound parameter, including the *MKI67* expression, and showed the highest average value in the PDGFRA subgroup (Figure S3). The distribution of the individual proliferation values in some subgroups was not gaussian, and especially for the EGFR↑ subgroup, two clusters could be separated, in correlation with *EGFR* overexpression values.

**Figure 2:**
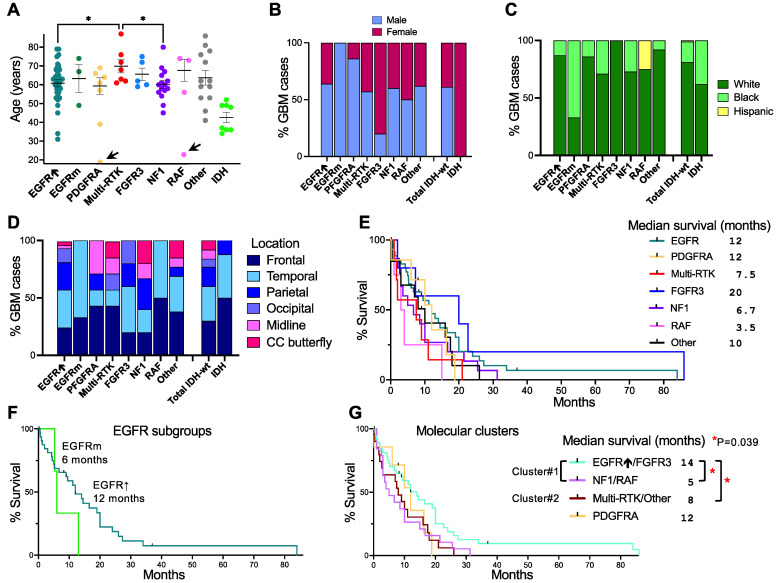
Demographic analysis of glioblastoma subgroups. (**A**) Mean ± SEM of individual age values excluding the outliers indicated by arrows. Statistically significant differences between IDH-wild-type subgroups are indicated by asterisks. (**B**) Sex distribution in glioblastoma subgroups. (**C**) Ethnic/race distribution in glioblastoma subgroups. (**D**) Tumor location distribution showing hemispheric and midline locations, the latter encompassing basal ganglia, pineal gland, and cerebellum. Corpus callosum (CC) symmetric or asymmetric butterfly glioblastoma is shown separately. (**E**–**G**) Kaplan–Meier survival curves for the 7 IDH-wild-type glioblastoma subgroups (**E**), separated EGFR subgroups (**F**), and molecular clusters (**G**). Median survival and statistical significance (*p*-values and asterisks) are indicated.

Hierarchical clustering showed the IDH subgroup separated from the IDH-wild-type subgroups, as expected (Figure 2G and Figure S4). Surprisingly, the EGFRm subgroup was also isolated from other subgroups. Another subgroup that segregated sharply from the rest was the PDGFRA subgroup. Unexpectedly, the EGFR↑ and NF1 subgroups clustered with the highest correlation coefficient, followed by the RAF subgroup. The FGFR3 subgroup more distantly clustered with the former three subgroups, whereas the Multi-RTK and Other subgroups formed a separate molecular cluster.

### 3.3. Glioblastoma Subgroup Demographic Characterization

The IDH-wild-type glioblastoma subgroups showed generally similar age central tendency parameters, with median and mean age values ranging from 60 to 62 and 59.3 to 63.7 years, respectively, for the most numerous subgroups, except for the Multi-RTK subgroup that showed significantly higher values, with a median of 68 and mean of 70 years (Figure 2A). The median and mean age for the IDH subgroup coincided, at 42 years, significantly lower than for the IDH-wild-type glioblastoma subgroups, aligning with other reports [2]. The male-to-female sex distribution of IDH-wild-type cases was 1.57:1, comparable with the reported ratio [2], but there was a bias towards 100% females in the IDH-mutant subgroup compared to the 0.96:1 reported ratio, probably at least partly due to the small sample (N = 8) (Figure 2B). The main deviations from the male-to-female ratio were noted in the PDGFRA and FGFR3 subgroups that showed bias towards males or females, respectively. The cohort comprised mainly Caucasian/White and African-American/Black patients, at a white-to-black ratio of 4.67:1 for IDH-wild-type glioblastoma and 1.7:1 for IDH-mutant glioblastoma (Figure 2C). Most IDH-wild-type subgroups had a similar ratio to the main group, except for the EGFRm subgroup which stood out with two African-American/Black out of three patients.

In the adult glioblastoma cohort, all except for one NF1 cerebellar tumor were supratentorial. This contrasted with the three histone H3-K27M-mutant DMG cases, of which two were spinal. The IDH-wild-type cases were evenly distributed between the frontal lobe (30%), temporal lobe (30%), and other locations, of which the parietal lobe was preponderant (17%) (Figure 2D). Midline locations comprised thalamus/internal capsule, pineal gland, and cerebellum, and were relatively rare, except in the PDGFRA subgroup that contained two tumors in the thalamus/internal capsule. Corpus callosum butterfly location was considered a separate location, as patients showing these tumors fare poorly, and isolated cases were seen in the major subgroups, with two cases clustered in the NF1 subgroup. In general, the location distribution varied among subgroups, with a preponderance of frontal cases in the PDGFRA, Multi-RTK, and IDH subgroups (Figure 2D).

The survival, as measured from the first surgery until death, in the IDH-wild-type glioblastoma cohort was 37.9% at 1 year, slightly lower than the reported one of 41% [1], and the median survival was 9.5 months. The best median survival was noted in the FGFR3 subgroup, at 20 months, followed by EGFR and PDGFRA, at 12 months (Figure 2E). The two longest-surviving patients, reaching 7 years, had tumors mapping to the FGFR3 and EGFR subgroups. The poorest median survival was observed in the RAF subgroup, at 3.5 months, followed by the NF1, Multi-RTK, and Other subgroups, at 6.7, 7.5, and 10 months, respectively. Further examination of the EGFRm subgroup showed a median survival of 6 months (Figure 2F). The molecular cluster #1 showed survival heterogeneity, with the EGFR↑ and FGFR3 subgroups showing longer median survival, compounded at 14 months, and the NF1 and RAF subgroups showing significantly shorter median survival (*p* = 0.039), compounded at 5 months (Figure 2G). The molecular cluster #2 composed of the Multi-RTK and Other subgroups showed also significantly lower survival than the EGFR↑/FGFR3 combined subgroups (*p* = 0.039), with a compound value at 8 months.

### 3.4. Enrichment of Histologic Patterns in Molecular Glioblastoma Subgroups

**Figure 3:**
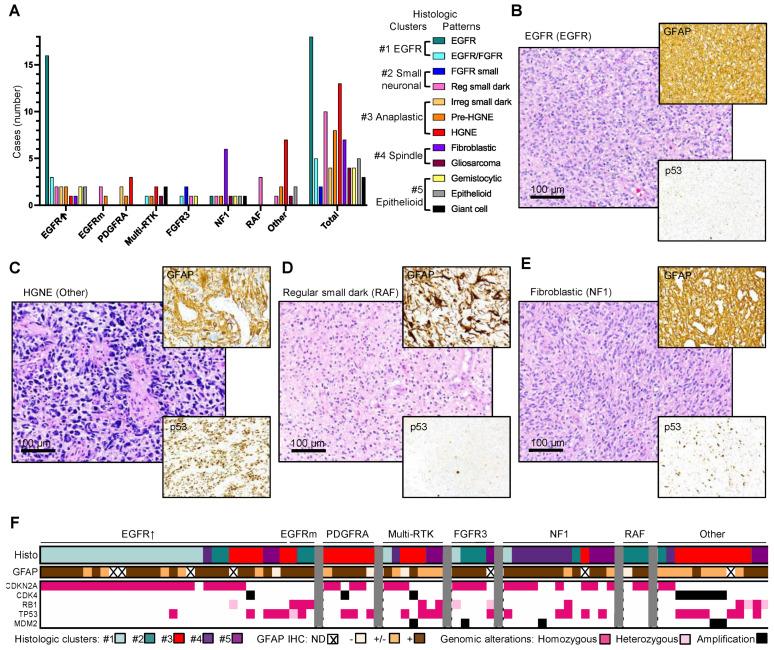
Histologic patterns and clusters in IDH-wild-type glioblastoma subgroups. (**A**) Bar graph showing histologic pattern distribution in the molecular glioblastoma subgroups and the total distribution in the cohort. Reg, regular nuclear contour; irreg, irregular nuclear contour; HGNE, high-grade neuroendocrine. (**B**–**E**) Representative H&E morphological appearances of the most common histological patterns. The molecular subgroup corresponding to the case is indicated in parenthesis. IHC with GFAP and p53 antibodies is shown in insets. (**F**) Histologic–molecular correlations. Histologic cluster distribution in individual cases from the IDH-wild-type glioblastoma subgroups shown in association with cell cycle G1 phase and p53 pathway mutations. The five histologic clusters (histo) are illustrated in (**A**).

The three WHO-recognized IDH-wild-type histologic variants, giant cell, gliosarcoma, and epithelioid [2], were scattered in the cohort at relatively low frequencies of 3.6%, 4.8%, and 6%, respectively (Figure 3A). The remaining samples were classified into nine additional patterns and further categorized on glioblastoma subgroups (Figure 3A). Four subgroups—EGFR, Other, NF1, and RAF—showed significant enrichment for a more specific pattern. The most frequent histologic pattern was seen in over half of the EGFR subgroup cases and appeared to also be specific, hence called “EGFR” (Figure 3A,B). It consisted of monomorphic cells with minimally discernible cytoplasm, blending in an eosinophilic extracellular matrix (ECM), with small, round, or slightly elongated nuclei with vesicular chromatin (Figure 3B, Figures S5 and S6). The second most frequent pattern had high-grade neuroendocrine (HGNE)/embryonal features previously described for the IDH#1 case [7], with pseudorosetting in most cases, and constituted about half of the PDGFRA and Other subgroup cases (Figure 3A,C, Figures S5 and S6). A similar pattern, called HGNE precursor (Pre-HGNE), had related cellular features to HGNE, except for a lack of nuclear molding, slightly vesicular chromatin, and lack of myxoid ECM (Figure S5). This pattern was also enriched in the PDGFRA and Other subgroups, but was also seen scattered in other subgroups. The third most common pattern was represented by cells with small, mostly round, hyperchromatic/dark nuclei with regular contours. It corresponded to all older adult RAF cases, where the nuclei were also surrounded by halos (Figure 3A,D, Figures S5 and S6). This pattern was also scattered in other subgroups and especially enriched in the EGFRm subgroup. Another histologic pattern that was almost entirely seen in the NF1 subgroup in approximately half of the cases was the fibroblastic type, with spindle cells embedded in eosinophilic or myxoid ECM (Figure 1A,D, Figures S5 and S6). The histology of the FGFR3 subgroup was described elsewhere [7] and, in the context of the entire cohort, showed overlap with EGFR, in a pattern called EGFR/FGFR, with a similar EGFR cell morphology pattern and prominent, intersecting capillary network (Figures S5 and S6). However, two FGFR3 cases constituting the FGFR small pattern appeared to show more specific nuclear characteristics, featuring small, finely stippled round or ovoid nuclei, and an ECM richer in hematoxylin-reacting components (Figures S5 and S6). As expected, the Multi-RTK subgroup showed a variation of patterns and included two of the three cases of giant cell glioblastoma from the cohort (Figures S5 and S6). These findings showed that the EGFR, PDGFRA, NF1, RAF, and Other subgroups were enriched in a defined histologic pattern, with the EGFR and fibroblastic patterns relatively specific for the EGFR↑ and NF1 subgroups, respectively.

To assess whether there were additional histologic–molecular associations, the 12 patterns were further clustered in five histologic clusters based on morphological similarities: #1/EGFR-like, #2/Small neuronal-like, #3/Anaplastic, #4/Spindle and #5/Epithelioid (Figure 3A,F). Case-by-case histologic cluster correlations with the most common mutations showed best correlations with cell cycle G1-phase mediators and *TP53* mutations. The EGFR-like histologic cluster was almost exclusively seen in the context of *CDKN2A/2B* homozygous loss and usually the absence of *TP53* mutations. *CDK4* mutations were only seen in the anaplastic cluster, usually associated with *TP53* or *MDM2* alterations. However, the anaplastic cluster was a feature of the PDGFRA subgroup, regardless of other alterations. The epithelioid cluster was associated with *TP53* mutations in 70% of cases, and with *RB1* mutations in 40% of the cases (Figure 3F).

GFAP IHC was performed for almost all cases, and showed reactivity in the majority of tumors, as expected (Figure 3F). The Multi-RTK, Other, and RAF subgroups showed lower or absent GFAP staining in 50%, 67%, and 71% of cases, respectively, suggesting lack of astrocytic differentiation in these subgroups.

## 4. Discussion

The WHO 2016 molecular subgrouping into IDH-wild-type and IDH-mutant glioblastoma emphasizes a significantly longer survival for the IDH-mutant subgroup, due to a slower tumor growth rate, and is reflected in a more insidious onset [21]. IDH-mutant cases represent approximately 10% of all glioblastoma cases [2] and 8% in this study. Except for a small number of cases, now classified as DMG with histone H3-K27M mutation, representing 4% in this study, there is no comprehensive histo-molecular classification for the IDH- and H3-wild-type glioblastoma cases approximating 90% of glioblastoma. Attempts have been made to distinguish histologic variants, and there are three variants of very rare incidence recognized in the WHO 2016 classification of brain tumors: gliosarcoma, giant cell, and epithelioid glioblastoma [2]. For the latter, the molecular association with *BRAF* p.V600E mutation in 50% of cases was noted [22]. Recent histologic–molecular correlations have also focused on the phenotype associated with *FGFR3* fusions in glioblastoma [7,23,24]. The efforts for molecularly classifying glioblastoma have been recently reviewed [25] and are based on a bioinformatics study categorizing in three different clusters the tumors with EGFR, NF1, and PDGFRA/IDH genetic alterations [26]. However, a more refined and inclusive molecular classification is warranted due to the lack of prognosis stratification for glioblastoma patients, and to the lack of efficacious specific therapies. In addition, developments in personalized medicine for other solid tumors and the availability of alternative targeted treatments for these demand a closer look at glioblastoma in order to design similar targeted therapies.

**Figure 4:**
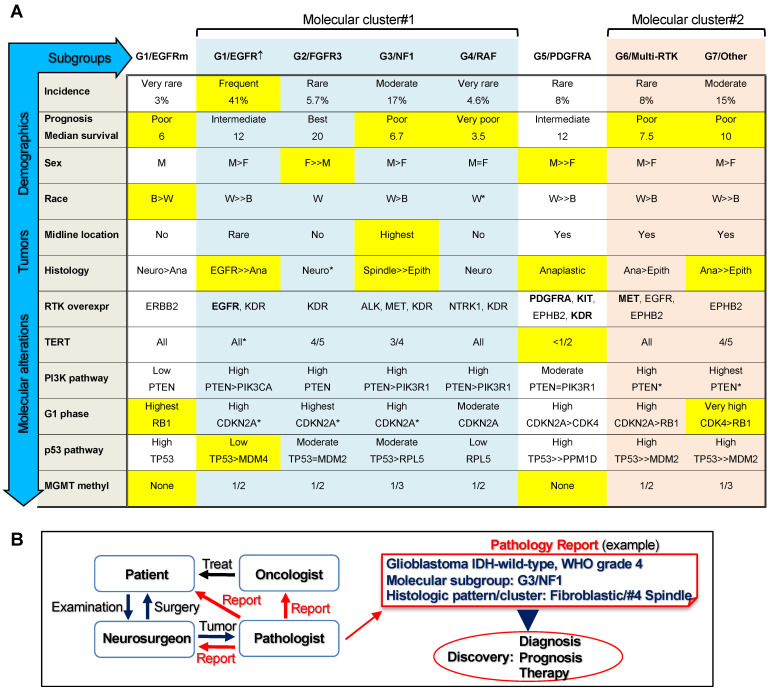
ERK/MAPK-based glioblastoma classification. (**A**) Multi-platform characterization of the IDH-wild-type glioblastoma G1–G7 molecular subgroups showing main demographic, histologic, RTK expression, and growth pathway mutation analysis: light blue and peach shading indicate molecular cluster#1 and #2, respectively; yellow shading highlights some characteristic features of the subgroup. M, male; F, female; B, African-American/Black; W, Caucasian/White. The midline location comprises all midline structures including the corpus callosum. Histologic clusters: Neuro, Small neuronal; Ana, Anaplastic; Epith, Epithelioid. RTK overexpression (overexpr) shows the most commonly upregulated RTKs; in bold are RTKs with gene amplifications. The mutation frequency is considered: highest, 100% cases; very high, >90% cases; high, ≥66.7% cases; moderate, between 33.3% and 66.7% cases; low, ≤33.3% cases. Asterisks (*) mark the presence of other minor components within the subgroup when only one parameter is shown. (**B**) Implementation of the glioblastoma classification in clinical practice and discovery. Schematic flowchart shows the management of the glioblastoma patient by the brain tumor team. An example of pathology report is shown, with incorporation of the glioblastoma molecular subgroup and histologic pattern and cluster.

This study represents a novel, stepwise, comprehensive classification of glioblastoma and includes pertinent genomic, transcriptomic, demographic, clinical, and histologic information (Figure 4A). It assigns all the glioblastoma cases to seven molecular subgroups, G1–G7, and shows their prognostic stratification. The classification was performed on a relatively large controlled cohort compared to cohorts from other studies [25]. Importantly, the cohort is representative for a mixed demographic population, including both Caucasian/White and African-American/Black ethnicities, providing thus more inclusive information than previously analyzed cohorts. The classification is based on my observation of the presence of non-redundant genomic alterations that activate RTKs and the upper segment of the MAPK/ERK pathway, whereas genomic alterations activating the PI3K pathway coexist with RTK alterations in a relatively even distribution (Figure 4A). The RTK subgroups G1/EGFR, G2/FGFR3, G5/PDGFRA, and G6/Multi-RTK accounted for roughly two-thirds of the glioblastoma IDH-wild-type cases, indicating a major role of RTKs in the pathogenesis of glioblastoma. An additional approximately 20% of cases were due to alteration in the upper segment of the ERK signaling pathway, namely NF1 tumor suppressor, in the subgroup G3/NF1, or RAF family members, in the subgroup G4/RAF. The remaining cases formed a separate subgroup, G7/Other, in which only genomic alterations of the PI3K pathway were apparent.

Hierarchical clustering analysis based on the main molecular characteristics of the subgroups showed two clusters and three independent subgroups (Figure 4A). The independent subgroups were the IDH, PDGFRA, and EGFRm. Whereas the IDH subgroup has been singled out in many studies because of its distinct better patient survival and lower tumor proliferation rate [21], the lack of clustering of PDGFRA and especially of EGFRm with other RTK subgroups is surprising. The main features of the IDH-wild-type glioblastoma subgroups are illustrated in Figure 4A. The G5/PDGFRA was the only subgroup that showed a majority of cases without *TERT* alterations as a mechanism of telomere elongation, an observation noted first by Higa et al. [27]. It also showed relatively few PI3K pathway alterations, equally divided between *PTEN* and *PIK3R1*. The histology was aggressive but the survival was not shorter than that for the G1/EGFR subgroup. The G1/EGFRm is a novel, very small subgroup, and will need further characterization to confirm the preliminary results presented in this study. It featured a shorter survival, the only predominant inclusion of African American/Black patients, a relatively aggressive histology, and *RB1* and *TP53* mutations, in contrast to the main G1/EGFR↑ subgroup.

The two clusters contained two or more subgroups. The largest subgroup, G1/EGFR↑, clustered closely with the G3/NF1 and G4/RAF subgroups, and more distantly with the G2/FGFR3 subgroup, in the molecular cluster#1. The G1/EGFR↑ subgroup showed the second-longest survival after the G2/FGFR3 subgroup, which was the subgroup with the longest survival, in concordance with a previous report [28]. The longest survivors of the cohort belonged to these two subgroups, with two survivors reaching 7 years, representing 2.2% of the IDH-wild-type cohort. Although the G3/NF1 subgroup alone showed similar survival relative to non-NF1 cases (not shown), as previously reported for the TCGA dataset [29], the combined G3/NF1-G4/RAF subgroups showed significantly shorter survival compared to the combined G1/EGFR↑-G2/FGFR3 subgroups, indicating a worse prognosis overall for patients with mutations in the upper segment of the ERK/MAPK pathway. The G2/FGFR3 subgroup was enriched in female patients, similar to the IDH subgroup, and in contrast to all other glioblastoma subgroups. Surprisingly, the histology of this subgroup was variable, with the small neuronal-like morphology as the most frequent. In contrast, both G1/EGFR and G3/NF1 subgroups showed a quasi-pathognomonic morphology in half of the tumors. All four subgroups from the molecular cluster #1 showed similar activation profiles of the major pathways: high incidence of PI3K pathway mutations, represented mainly by *PTEN* but also by *PIK3CA* and *PIK3R1* mutations, high incidence of *CDKN2A* homozygous loss coupled with *CDK6* overexpression, and conversely, low incidence of *TP53* mutations.

The molecular cluster#2 comprised cases with similar demographic and histologic characteristics, including relatively poor survival, classified separately into the G6/Multi-RTK and G7/Other subgroups. The molecular signature was also similar for the PI3K and p53 pathways, with a high incidence of *PTEN* and *TP53* mutations, but showed clustering of *CDK4* amplification in the G7/Other subgroup. Although the G6/Multi-RTK subgroup appeared heterogeneous, with concurrent genomic alterations and overexpression of a wide range of RTKs, it may represent a good target for the multi-RTK therapy that has shown some success in recurrent glioblastoma [30].

This classification required both genomic and transcriptomic information. The transcriptomic analysis uncovered pathogenic fusions with subgrouping relevance, and examined the translation of CN alterations into gene expression levels, as we have previously shown that gene amplification does not always result in mRNA overexpression [7]. The landscaping of RTK expression and correlation with genomic alterations has not been performed previously in glioblastoma. Its major findings are (1) lack of overlap between *EGFR*, *PDGFRA*, and *FGFR3* alterations, including overexpression; (2) good correlation between *EGFR* amplification and high overexpression in the vast majority of cases; a similar correlation for *MET* and *FGFR2*-amplified cases was also found; (3) presence of a small subgroup characterized by *EGFR* activating mutations without gene amplification or high overexpression; (4) lack of correlation between *PDGFRA* locus amplification, including *KIT* and *KDR*, and overexpression, requiring correct assignment of cases with high *PDGF*

*RA* overexpression in the G5/PDGFRA subgroup and of the *PDGFRA*-amplified non-overexpressing cases in the G6/Multi-RTK subgroup; (5) *KDR* overexpression in the majority of cases and at higher levels in the molecular cluster#1, most likely in the vascular compartment; (6) overexpression without amplification of additional RTKs in many cases, such as *NTRK1*, *ERBB2*/*ERBB3*, *EPHA3*/*EPHB2* and *ALK*, some showing subgroup specificity (Figure 4A).

The RTK landscaping, pathway associations, and subgrouping efforts presented here also carry a major impact for the clinical management of glioblastoma, including diagnosis, prognosis, and therapy (Figure 4B). Although many clinical trials targeting a plethora of pathways are ongoing in glioblastoma [31] and aim at the major pathways presented here, correct patient inclusion is crucial for regimen success. For example, the G1/EGFRm subgroup may represent a more promising target to EGFR inhibitors than the larger G1/EGFR↑ subgroup, similarly to non-small cell lung carcinomas with *EGFR* mutations. Likewise, PDGFRα inhibitors may not work for tumors with *PDGFRA* amplification without overexpression. Specific RTK inhibitors are available not only for the RTKs with genomic alterations for which drug trials are usually designed, such as EGFR, PDGFRα, FGFR2 and FGFR3, NTRK1, and MET, but also for RTKs with overexpression with or without mutations of unknown significance, such as ALK, ERBB2 (Her2/Neu), and EPHA3 [32]. Together with new targets revealed in this study, such as EPHB2, these RTKs also warrant consideration for glioblastoma therapy. Moreover, efforts should include RTK family or multi-RTK strategies to cover convergent growth signaling from multiple RTKs, and testing for RTK reprogramming leading to drug resistance [14,33]. Open questions remain, such as the use of combination therapy for targeting downstream or parallel growth pathways. Of these, the PI3K pathway may represent a selective target for the tumors composing the G7/Other subgroup that apparently rely predominantly on this canonical growth pathway.

## 5. Conclusions

In conclusion, I presented here a comprehensive multi-platform glioblastoma classification with large patient inclusion and immediate field applicability for diagnosis and prognosis (Figure 4). The incorporation of this novel classification in the pathology report will foster discovery by immediate molecular subgroup stratification, data sorting, and personalized follow-up of patients. This classification complements and expands the previous efforts for a better understanding of this deadly disease, and lays the foundation for precision therapy design.

## Figures and Tables

**Table 1:** Mutation percent (%) frequency in glioblastoma subgroups.

Gene	Total IDH wt *n* = 87	EGFR ↑ *n* = 33	EGFR m *n* = 3	PDGFRA *n* = 7	Multi-RTK *n* = 7	FGFR3 *n* = 5	NF1 *n* = 15	RAF *n* = 4	Other *n* = 13	IDH m *n* = 7
TERT ^1^	85 (81.2)	96.4	**100**	42.9	**100**	80	73.3	**100**	81.8	28.6
PTEN	53	48.5	33.3	28. 6	71.4	80	46. 7	50	69.2	0
PIK3CA	18.4	**27.3**	0	0	14.3	0	13.3	0	15.4	**28.6**
PIK3R1	12.6	9.1	0	**28.6**	0	0	26.7	25	7.7	0
PI3K/mTOR ^2^	75.9	75.8	33.3	42.9	71.4	80	80	75	**100**	42.9
CDKN2A ↓	55.2	72.7	0	57.1	42.9	**80**	60	50	15.4	42.9
CDK4 ↑	11.5	3	0	28.6	14.3	0	0	0	**46.2**	14.3
RB1	12.6	0	**100**	0	28.6	20	6.7	0	30.8	0
G1 phase ^3^	79.3	75.8	**100**	85.7	85.7	**100**	66.7	50	92.3	57.1
TP53	33.3	18.2	**66.7**	57.1	57.1	20	33.3	0	53.8	**100**
MDM2 ↑	5.7	0	0	0	14.3	**20**	6.7	0	15.4	0
MDM4 ↑	4.6	**9.1**	0	0	0	0	0	0	7.7	0
RPL5	5.7	6.1	0	0	0	0	**13.3**	25	0	0
PPM1D	1.1	0	0	**14.3**	0	0	0	0	0	0
TP53 path ^4^	49.4	33.3	66.7	**71.4**	**71.4**	40	53.3	25	69.2	**100**
ATM	12.6	12.1	0	**42.9**	0	20	13.3	25	0	0
BRCA2	5.7	9.1	**33.3**	14.3	0	0	0	0	0	28.6
MMR ^5^	11.5	12.1	0	28.6	14.3	**40**	6.7	0	0	14.3
DDR path ^6^	26.4	27.3	33.3	**71.4**	14.3	60	20	25	0	42.9
STAG2	12.6	15.2	0	14.3	14.3	20	13.3	0	0	0
SWI/SNF ^7^	13.8	15.2	0	14.3	28.6	0	20	25	0	**57.1**
Other ChRm ^8^	32.2	30.3	0	14.3	57.1	**100**	20	0	38.5	14.3
MGMT methyl	36.1	43.3	0	0	**50**	**50**	27.3	**50**	36.4	42.9

Wt, wild-type; ↑, gene amplification; ↓, homozygous CN loss; m, point mutation; path, pathway; DDR, DNA damage response; MMR, mismatch repair; ChRm, chromatin remodeling; methyl, methylation. The highest % for a certain alteration is indicated in bold. ^1^ % cases with cumulated *TERT* promoter mutation and *TERT* overexpression in the absence of mutation. The value in brackets corresponds to the total % cases with *TERT* promoter mutations only. ^2^ % cases with at least one alteration in *PTEN*, *PIK3CA*, *PIK3R1*, *TSC2*, or *MTOR*. ^3^ % cases with *CDKN2A* homozygous loss, *CDK4* amplification, or *RB1* mutation. ^4^ % cases with *TP53, MDM2*, *MDM4*, *RPL5*, and *PPM1D* alterations. ^5^ % cases with either *MSH6*, *MSH5*, *PMS2*, or *MLH3* pathogenic mutations. ^6^ % cases with at least one alteration in *ATM*, *BRCA2*, or MMR genes. ^7^ % cases with SWI/SNF complex *ARID1A*, *ARID1B*, *ARID2*, *SMARCA1*, *SMARCA4,* or *PBRM1* pathogenic mutations. ^8^ % cases with either *YEATS4* amplification or *DNM3TA*, *TET2*, *EZH2*, *SUZ12*, *ASXL1*, *ASXL2*, *KDM5C*, *KDM6A*, *KMT2C*, *KMT2D,* or *CREBBP* pathogenic mutations.

## Data Availability

Supporting data for this manuscript are available in the Supplementary Material and upon request to the corresponding author. Physicians may also contact the author for hands-on help in reporting the glioblastoma subgroups.

## Supplementary Materials

The following are available online at https://www.mdpi.com/article/10.3390/cancers13184532/s1, Figure S1: Glioblastoma subgroups—landscape of RTK expression, Figure S2: Glioblastoma subgroups—tumor mutation burden, Figure S3: Glioblastoma subgroups—proliferation, Figure S4: Glioblastoma subgroups—correlation matrix, Figure S5: Glioblastoma IDH-wild-type—characteristic nuclear morphology of the histologic patterns, Figure S6: Glioblastoma IDH-wild-type—correspondence between histologic patterns and molecular subgroups, Table S1: Glioblastoma genomic alterations, Table S2: RTK classes and genes.

## Abbreviations

C-	carboxyl
CN	copy number
DDR	DNA damage response
ECM	extracellular matrix
EGFR	epidermal growth factor receptor; EGFR↑, EGFR with gene amplification with or without other genetic alterations; EGFRm, EGFR with gene mutation only
ERK/MAPK	extracellular signal-regulated kinase/mitogen-activated protein kinase
FFPE	formalin-fixed paraffin-embedded
FGFR	fibroblast growth factor receptor
GFAP	glial fibrillary acidic protein
H&E	hematoxylin eosin
IDH	isocitrate dehydrogenase
IHC	immunohistochemistry
LOH	loss of heterozygosity
NF1	neurofibromatosis type 1
NGS	next generation sequencing
PDGFR	platelet-derived growth factor receptor
PI3K	phosphatidylinositol 3-OH kinase
RTK	receptor tyrosine kinase
WHO	World Health Organization
