# Supplementary material for: Multi-Platform Classification of IDH-Wild-Type Glioblastoma Based on ERK/MAPK Pathway: Diagnostic, Prognostic and Therapeutic Implications
Source: Cancers (Basel). 2021 Sep 9;13(18):4532. doi: 10.3390/cancers13184532 (PMC8470497; doi:10.3390/cancers13184532)
Supplement: Supplementary file 1 [file cancers-13-04532-s001.zip › cancers-1328061-supplementary.pdf]

SUPPLEMENTAL FIGURES S1-S6

S1: Glioblastoma molecular subgroups: landscape of RTK expression
S2: Glioblastoma molecular subgroups: tumor mutation burden
S3: Glioblastoma molecular subgroups: proliferation
S4: Glioblastoma molecular subgroups: correlation matrix
S5: Glioblastoma IDH-wild-type: characteristic nuclear morphology of the histologic patterns
S6: Glioblastoma IDH-wild-type: correspondence between histologic patterns and molecular subgroups

SUPPLEMENTAL TABLES S1-S2

S1: Glioblastoma genomic alterations
S2: RTK classes and genes

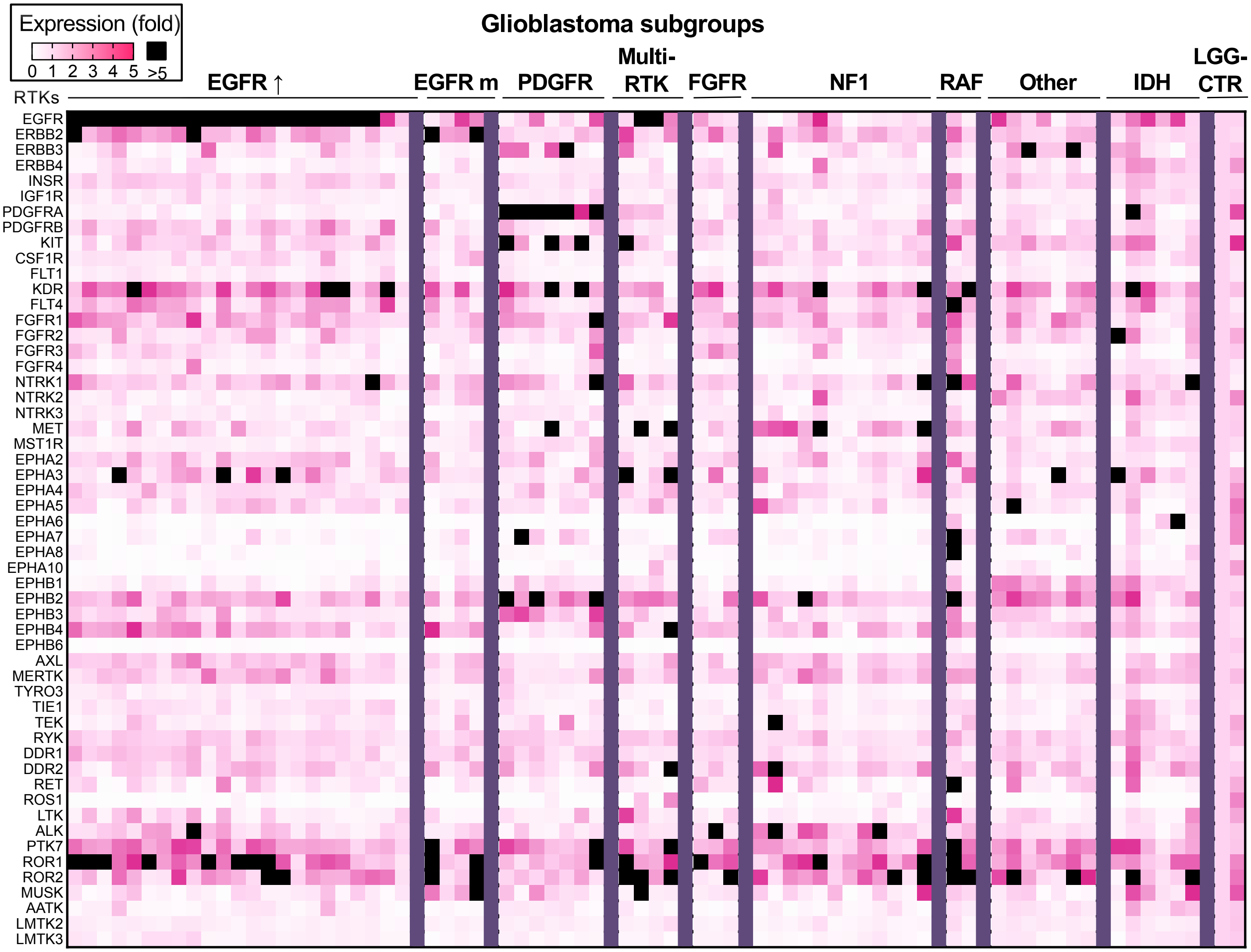

Supplemental Figure S1. **Landscape of RTK expression in glioblastoma subgroups.** RTK fold-expression relative to precursor low-grade glioma control (LGG-CTR) showing only the RTKs with raw values >500 in at least one sample (see Materials and Methods for RNA expression analysis). EGFR↑, *EGFR* amplification; EGFRm, *EGFR* point mutation. Note strong upregulation of *EGFR* expression in almost all cases with *EGFR* amplification. Note also increased expression of various RTKs in the absence of genetic alteration in some of the samples.

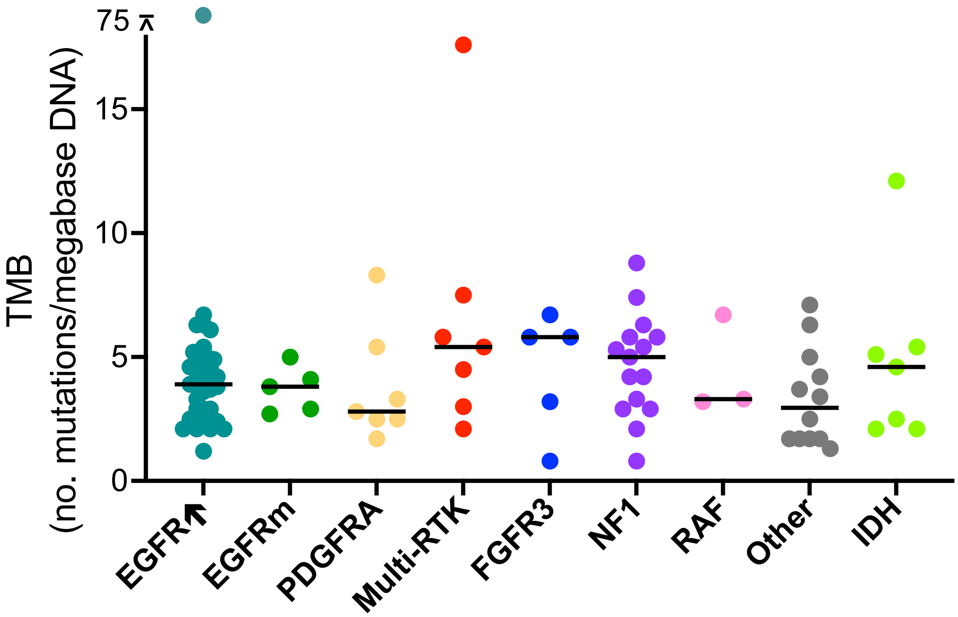

Supplemental Figure S2. <u>Tumor mutation burden (TMB) in glioblastoma subgroups</u>. Plot of individual TMB values medians indicated by horizontal bars. In the EGFRm subgroup, the samples from multifocal cases with EGFR mutations without amplification were added to the analysis. All values are from tumors in the absence of any treatment. Note only three tumors with TBM>10, and only three subgroups with median values >5.

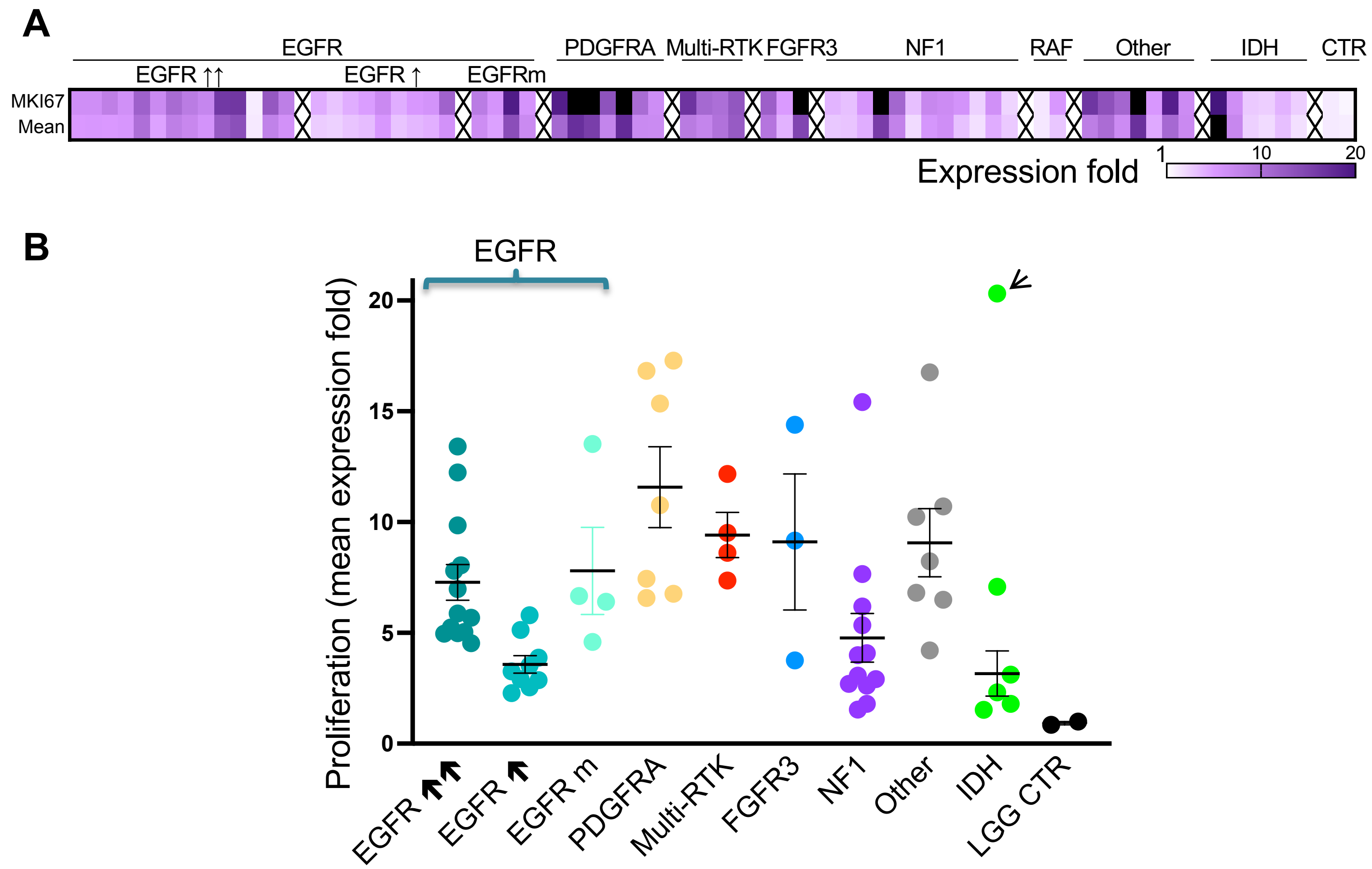

Supplemental Figure S3. **Glioblastoma subgroups proliferation.** **A.** Expression analysis heatmap of *MIK67* (shown), *TYMS*, *CCNB2*, *CDK1*, *CHEK1* and *EZH2* yielding a mean expression index, as fold overexpression relatively to precursor low-grade glioma controls (LGG). The sample order is the same as in Figure 1. Note separation of the EGFR subgroup in three proliferation clusters: with amplification and high overexpression (↑↑), with amplification and medium overexpression (↑), and with mutation only. The EGFR#12 tumor shows very low values due to recent prior radiation, and was excluded from further analysis. **B.** Mean±SEM plot of individual proliferation values from (A). Note different proliferation indices in the three EGFR subgroups. The outlier value from the IDH subgroup indicated by arrow corresponds to IDH#1 and was excluded from the mean calculation.

	EGFR↑	EGFRm	PDGFRA	Multi-RTK	FGFR3	NF1	RAF	Other	IDH
**EGFR↑**	1.00	0.36	0.62	0.79	0.79	0.89	0.88	0.68	0.38
**EGFRm**	0.36	1.00	0.35	0.62	0.30	0.40	0.34	0.62	0.26
**PDGFRA**	0.62	0.35	1.00	0.51	0.54	0.56	0.49	0.50	0.40
**Multi-RTK**	0.79	0.62	0.51	1.00	0.79	0.80	0.71	0.88	0.45
**FGFR3**	0.79	0.30	0.54	0.79	1.00	0.69	0.62	0.65	0.16
**NF1**	0.89	0.40	0.56	0.80	0.69	1.00	0.87	0.72	0.40
**RAF**	0.88	0.34	0.49	0.71	0.62	0.87	1.00	0.63	0.22
**Other**	0.68	0.62	0.50	0.88	0.65	0.72	0.63	1.00	0.34
**IDH**	0.38	0.26	0.40	0.45	0.16	0.40	0.22	0.34	1.00

Supplemental Figure S4. Glioblastoma subgroups correlation matrix. Pearson correlation coefficient between each pair of subgroups is shown. Closest correlations are indicated with white labeling on blue background. Note unique profiles for the IDH, EGFRm and PDGFRA subgroups.

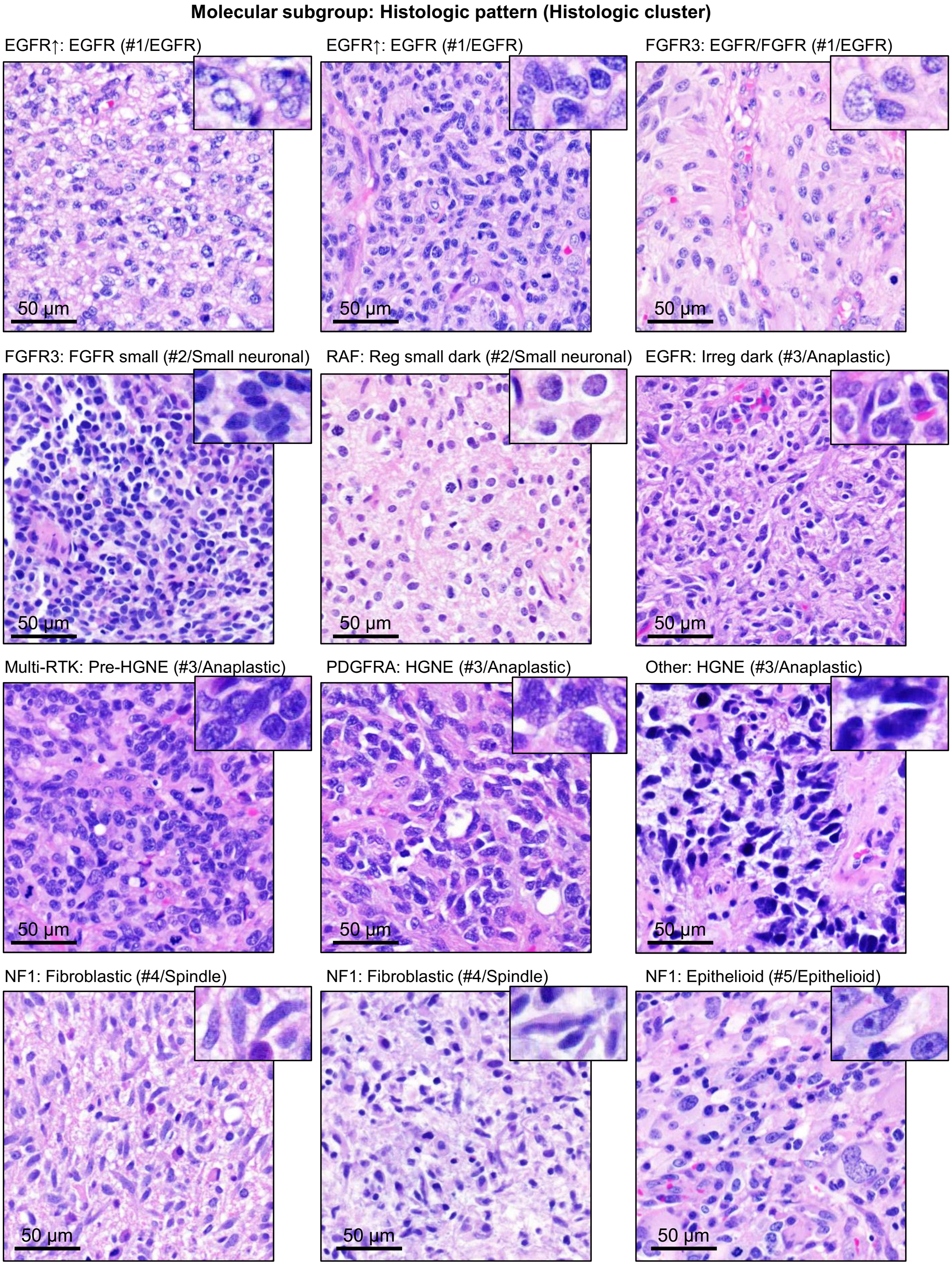

Supplemental Figure S5. **Characteristic nuclear morphology of the glioblastoma histologic patterns.** The nuclear morphology is detailed for the representative histologic patterns seen in the IDH-wild-type glioblastoma molecular subgroups, as labeled on top of the figure. H&E images were acquired at 40x magnification, and all panels and all insets are shown at the same magnification, respectively. Note characteristic small nuclei with open vesicular chromatin in EGFR-like cluster#1 tumors, small, regular nuclei with finely stippled or "salt-and-pepper" chromatin in Small neuronal-like cluster#2 tumors, hyperchromatic, irregular nuclei in Anaplastic cluster#3 tumors, spindle nuclei in Spindle cluster#4 tumors, and centrally-placed nuclei with conspicuous nucleolus in the epithelioid pattern of Epithelioid cluster#5 tumors.

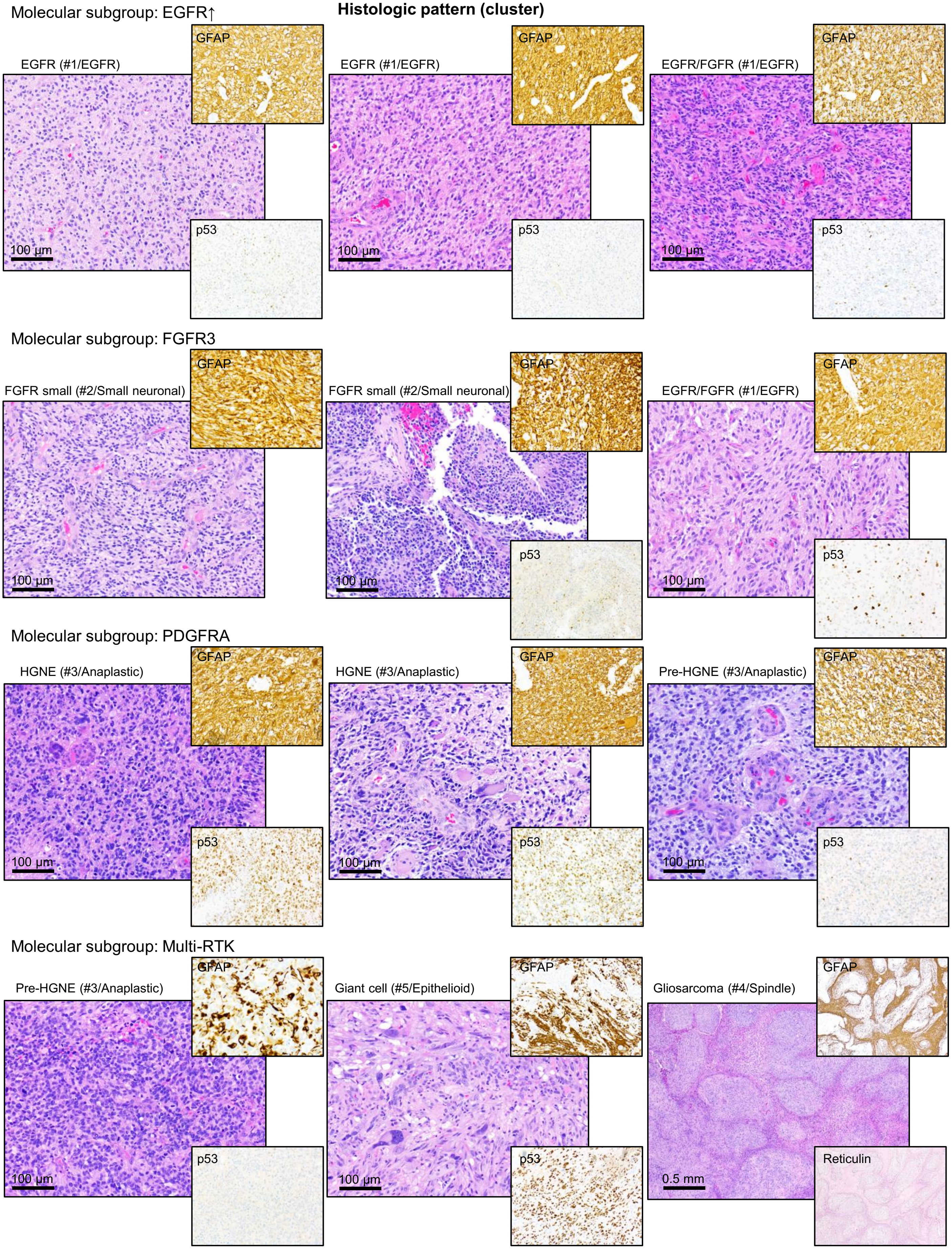
Molecular subgroup: EGFR↑
Histologic pattern (cluster)
EGFR (#1/EGFR)
GFAP
p53
100 µm
EGFR (#1/EGFR)
GFAP
p53
100 µm
EGFR/FGFR (#1/EGFR)
GFAP
p53
100 µm
Molecular subgroup: FGFR3
FGFR small (#2/Small neuronal)
GFAP
100 µm
FGFR small (#2/Small neuronal)
GFAP
p53
100 µm
EGFR/FGFR (#1/EGFR)
GFAP
p53
100 µm
Molecular subgroup: PDGFRA
HGNE (#3/Anaplastic)
GFAP
p53
100 µm
HGNE (#3/Anaplastic)
GFAP
p53
100 µm
Pre-HGNE (#3/Anaplastic)
GFAP
p53
100 µm
Molecular subgroup: Multi-RTK
Pre-HGNE (#3/Anaplastic)
GFAP
p53
100 µm
Giant cell (#5/Epithelioid)
GFAP
p53
100 µm
Gliosarcoma (#4/Spindle)
GFAP
Reticulin
0.5 mm

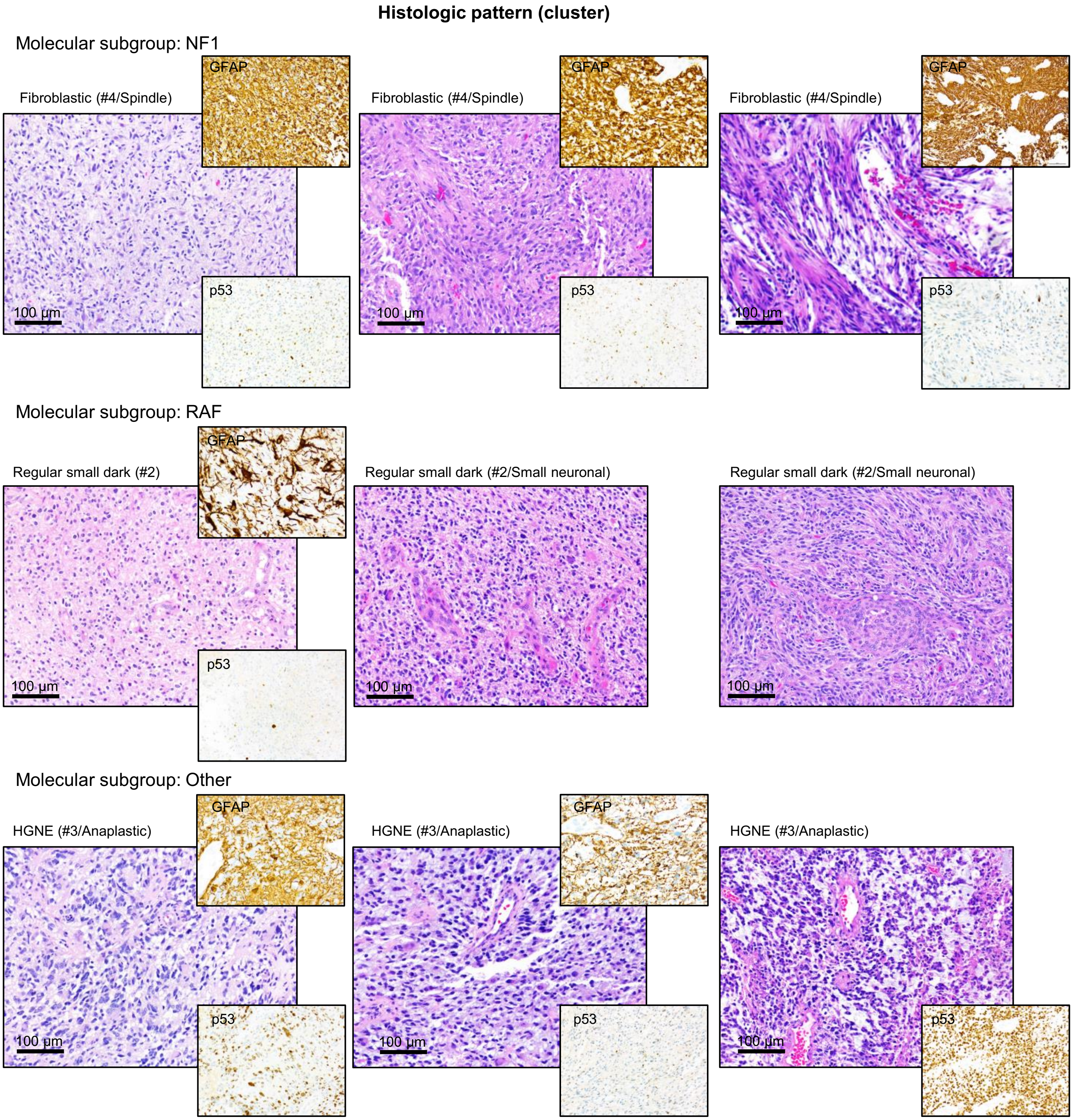

Supplemental Figure S6. <u>Correspondence between histologic patterns and molecular subgroups in IDH-wild-type glioblastoma</u>. This figure is displayed on two pages and shows three examples of representative histologic patterns for each of the IDH-wild-type glioblastoma molecular subgroups. H&E images were acquired at 20x magnification, as described in Materials and Methods. Corresponding IHCs for GFAP and p53 are also shown in insets. The images show the characteristic morphology of tumors from 21 different patients.

Supplemental Table S1. RTK-MAPK pathway genetic alterations

Colored font: recurrent mutations
Bold font: di-sulfide bond mutations
Fold overexpression from whole transcriptomics
* Unless otherwise mentioned, the mutations are clonal

Case	Gene	Amplif	Fold	Mutation/fusion	Amino aci	Effect	Associations*	NM
EGFR#1	EGFR	Yes	72					_005228
EGFR#2	EGFR	Yes	56	Exons 2-7		Splice	vIII	_005228
EGFR#3	EGFR	Yes	46	c.1951G>A	V651M	Missense	w/ amplificatior	_005228
EGFR#4	EGFR	Yes	43	**c.1907G>T**	**C636F**	Missense	w/ amplificatior	_005228
	EGFR	Yes		EGFR-SEPT14		In-frame	w/ amplificatior	_005228
EGFR#5	EGFR	Yes	42					_005228
EGFR#6	EGFR	Yes	37	c.866C>T	A289V	Missense		_005228
	EGFR	Yes		Exons 25-27		Splice	vIVa	_005228
EGFR#7	EGFR	Yes	34	c.323G>A	R108K	Missense	w/ amplificatior	_005228
EGFR#8	EGFR	Yes	30	EGFR-SEPT14		In-frame	w/ amplificatior	_005228
	EPHB1	No		c.2138A>G	D713G	Missense		_004441
EGFR#9	EGFR	Yes	29	Exons 2-7		Splice	vIII	_005228
EGFR#10	EGFR	Yes	26					_005228
EGFR#11	EGFR	Yes	26					_005228
EGFR#12	EGFR	Yes	25					_005228
EGFR#13	EGFR	Yes	25	EGFR-SEPT14		In-frame	w/ amplificatior	_005228
EGFR#14	EGFR	Yes	24	Exons 2-7		Splice	vIII	_005228
	EGFR	Yes		EGFR-VOPP1		In-frame	w/ amplificatior	_005228
EGFR#15	EGFR	Yes	18	Exons 25-27		Splice	vIVa	_005228
	ALK	No		c.3402G>C	Q1134H	Missense		_004304
EGFR#16	EGFR	Yes	13	Exons 2-7		Splice	vIII	_005228
	ERRFI1			CN loss			Homozygous	
	ALK	No		c.502T>C	F168L	Missense	Likely germline	_004304
EGFR#17	EGFR	Yes	12	c.866C>T	A289V	Missense		_005228
	EGFR	Yes		Exons 25-28		Deletion	C-terminal	_005228
	ALK	No		c.3587T>A	L1196Q	Missense		_004304
EGFR#18	EGFR	Yes	10	**c.884G>A**	**C295Y**	Missense		_005228
EGFR#19	EGFR	Yes	9.4	Exons 2-7		Splice	vIII	_005228
EGFR#20	EGFR	Yes	7.8	Exons 2-7		Splice	vIII	_005228
	EGFR	Yes		CTDSP2-EGFR		In-frame	Fusion	_005228
EGFR#21	EGFR	Yes	5.8	SEC61G-EGFR		In-frame	Fusion	_005228
EGFR#22	EGFR	Yes	4.5	EGFR-SEPT14		In-frame	w/ amplificatior	_005228
EGFR#23	EGFR	Yes	1.4					_005228
EGFR#24	EGFR	Yes		c.1793G>T	G598V	Missense	w/ amplificatior	_005228
EGFR#25	EGFR	Yes						_005228
EGFR#26	EGFR	Yes		c.866C>T	A289V	Missense		_005228

EGFR#27	EGFR	Yes		c.323G>A	R108K	Missense	w/ amplificatior	_005228
EGFR#28	EGFR	Yes		**c.976T>C**	**C326R**	Missense	w/ amplificatior	_005228
EGFR#29	EGFR	Yes		c.787A>C	T263P	Missense	w/ amplificatior	_005228
	EGFR	Yes		c.1793G>T	G598V	Missense		_005228
EGFR#30	EGFR	Yes		**c.932G>A**	**C311Y**	Missense	w/ amplificatior	_005228
EGFR#31	EGFR	Yes						_005228
EGFR#32	EGFR	Yes		Exon 20		Insertion		_005228
EGFR#33	EGFR	Yes		c.1088C>T	T363I	Missense	w/ amplificatior	_005228
EGFR#34	EGFR	No	1.5	c.874G>T	V292L	Missense		_005228
	PDGFRA	No		c.2930T>G	V977G	Missense	Germline	_006206
EGFR#35	EGFR	No	4.3	**c.1773C>G**	**C591W**	Missense		_005228
EGFR#36	EGFR	No	2.8		P596L	Missense		_005228
	EGFR	No		c.1280G>A	R427H	Missense		_005228
	EPHA8	No		c.181G>A	D61N	Missense		_020526
PDGFRA#1	PDGFRA	Yes	25	**c.704G>A**	**C235Y**	Missense		_006206
	KIT	Yes	5.4					
	KDR	Yes	4.9					_002253
PDGFRA#2	PDGFRA	Yes	22	c.1607T>A	V536E	Missense		_006206
	EPHB2	No	14	c.2375G>A	W792*	Nonsense	Germline	_001309193
PDGFRA#3	PDGFRA	Yes	15	**c.704G>T**	**C235F**	Missense	w/ amplificatior	_006206
PDGFRA#4	PDGFRA	Yes	15					_006206
	KIT	Yes	21					
	KDR	Yes	10					_002253
	MET	No	27	PTPRZ1-MET		In-frame	Fusion	_000245
PDGFRA#5	PDGFRA	No	9.8	c.925G>C	V309L	Missense	w/ overexpress	_006206
PDGFRA#6	PDGFRA	Yes	4.9					_006206
	KIT	Yes	7					
	KDR	Yes	10					_002253
	LZTR1			c.321-8_324del	R107fs	Frameshift		_006767
	ARAF	No		c.83A>C	K28T	Missense		_001654
PDGFRA#7	PDGFRA	Yes	26	c.932-1_934delGAGA	K312del	Splice	w/ overexpress	_006206
	H3F3A	No			G34R	Missense		_002107
	IDH2	No			G190S	Missense	Likely germline	_02168
Multi-RTK#1	PDGFRA	Yes	1.8					_006206
	KIT	No	13					
	FGFR2	No		c.1501G>A	A501T	Missense	Subclonal	_022970
	NRAS	No		c.181C>A	Q61K	Missense	Subclonal	_002524
Multi-RTK#2	PDGFRA	Yes	1.5					_006206
	KIT	Yes	1.4					
	MET	Yes	39					_000245
	EGFR	No	9.6					_005228
Multi-RTK#3	PDGFRA	Yes	1.6					_006206
	EGFR	No	6.2					_005228

Multi-RTK#4	EPHA8	No		c.593G>A	R198H	Missense		_020526
	EPHB1	No		c.1817C>T	A606V	Missense		_004441
	MET	No	28					_000245
	MSH6	No		c.2615_2616dup	G873fs	Frameshift	Germline	_000179
Multi-RTK#5	MET	Yes	very high					_000245
Multi-RTK#6	ERRFI1			CN loss			Homozygous	
	FGFR2	No		c.252G>T	R84S	Missense	Likely germline	_022970
	PTPN11			c.181G>A	D61N	Missense	Subclonal	_002834
	LZTR1			CN loss			Homozygous	_006767
Multi-RTK#7	NTRK1	No		LMNA-NTRK1				
	FGFR3	No		C.505G>A	A169T	Missense	Subclonal	_000142
FGFR#1	FGFR3	No		FGFR3-TACC3		In-frame	Fusion	_000142
	FRS2	Yes	44					
FGFR#2	FGFR3	No		FGFR3-TACC3		In-frame	Fusion	_000142
FGFR#3	FGFR3	No		FGFR3-TLN1		In-frame	Fusion	_000142
FGFR#4	FGFR3	No		c.2274+26_2346dup	S782ins(7	Insertion	C-terminal	_000142
	FRS2	Yes						
FGFR#5	FGFR3	No		c.1672G>A	A558T	Missense		_000142
NF1#1	NF1			c.5977C>T	Q1993*	Nonsense	LOH	_001042492
	PDGFRA	No		c.853A>G	S285G	Missense	Germline	_006206
NF1#2	NF1			c.4790G>T	G1597V	Missense	Germline; LOH	_001042492
NF1#3	NF1			c.8033T>G	L2678*	Nonsense	LOH	_001042492
NF1#4	NF1			c.499_502delTGTT	C167fs	Frameshift	LOH	_001042492
NF1#5	NF1			c.7549C>T	R2517*	Nonsense	LOH	_001042492
	PTPN11			c.215C>T	A72V	Missense	Subclonal	_002834
	PTPN11			c.226G>C	E76Q	Missense	Subclonal	_002834
NF1#6	NF1			c.5797G>T	G1933*	Nonsense		_001042492
	NF1			c.6390dup	H2131fs	Frameshift		_001042492
NF1#7	NF1			c.6852_6855del	Y2285fs	Frameshift	LOH	_001042492
	PDGFRA	No		c.3149T>C	I1050T	Missense	Likely germline	_006206
NF1#8	NF1			c.7897G>T	E2633*	Nonsense		_001042492
	NF1			NF1-NUDT12	S1935fs	Frameshift	Fusion	_001042492
	PTPN11			c.1520C>A	T507K	Missense		_002834
NF1#9	NF1			c.5032_5034dupTAT	Y1678du	Insertion	Germline	_001042492
	NF1			c.1020dupT	V341fs	Frameshift		_001042492
	PTPN11			c.205G>A	E69K	Missense	Subclonal	_002834
	LZTR1			c.321-8_324del	R107fs	Frameshift	LOH	_006767
	FRS2	Yes	3.7					
NF1#10	NF1			c.6007-1G>A		Splice		_001042492
	NF1			c.1381C>T	R461*	Nonsense		_001042492
	ERBB4	No		c.2539C>T	R847C	Missense	Likely germline	_005235
NF1#11	NF1			c.4600C>T	R1534*	Nonsense		_001042492
	NF1			c.4625T>C	L1542P	Missense		_001042492

NF1#12	NF1			NF1-chr2p15	G1597fs	Frameshift	Fusion	_001042492
NF1#13	NF1			c.4267G>T	E1423*	Nonsense		_001042492
NF1#14	NF1			c.479G>A	R160K	Splice	LOH	_001042492
NF1#15	NF1			c.6772C>T	R2258*	Nonsense	LOH	_001042492
	PTPN11			c.417G>C	E139D	Missense		_002834
RAF#1	BRAF	No		c.1799T>A	V600E	Missense		_004333
RAF#2	BRAF	No		c.1780G>A	D594N	Missense		_004333
RAF#3	BRAF	No		c.1447A>G	K483E	Missense		_004333
RAF#4	RAF1	Yes		c.770C>T	S257L	Missense	w/ overexpress	_002880
IDH#1	IDH1			c.395G>A	R132H	Missense		_001282387
	FGFR2	Yes	68					_022970
IDH#2	IDH1			c.395G>A	R132H	Missense		_001282387
IDH#3	IDH1			c.395G>A	R132H	Missense		_001282387
IDH#4	IDH1			c.395G>A	R132H	Missense		_001282387
IDH#5	IDH1			c.395G>A	R132H	Missense		_001282387
IDH#6	IDH1			c.395G>A	R132H	Missense		_001282387
IDH#7	IDH1			c.395G>A	R132H	Missense		_001282387
IDH#8	IDH1				R132H	Missense		_001282387

Supplemental Table S2. Human RTK classes and genes.

Class	Family name	Members (proteins)	Members (genes)
I	EGFR	EGFR, ERBB2-4	Same
II	Insulin receptor	INSR, IGFR	INSR, IGF1R
III	PDGFR	PDGFRα, PDGFRβ, KIT, M-CSFR, FLT3	PDGFRA, PDGFRB, KIT, CSF1R, FLT3
IV	VEGFR	VEGFR1-3	FLT1, KDR, FLT4
V	FGFR	FGFR1-4	Same
VI	CCK	CCK4	PTK7 (inactive; WNT pathway)
VII	NGFR	TRKA-C	NTRK1-3
VIII	HGFR	MET, RON	MET, MST1R
IX	EPHR	EPHA1-8, EPHA10, EPHB1-4, EPHB6	Same
X	AXL	AXL, MER, TYRO3	AXL, MERTK, TYRO3
XI	TIE	TIE, TEK	TIE1, TEK
XII	RYK	RYK	Same
XIII	DDR	DDR1-2	Same
XIV	RET	RET	Same
XV	ROS	ROS	ROS1
XVI	LTK	LTK, ALK	Same
XVII	ROR	ROR1-2	Same; (pseudokinases; WNT pathway)
XVIII	MUSK	MUSK	Same
XIX	LMR	AATYK1-3	AATK, LMTK2, LMTK3

EGFR, epidermal growth factor receptor; PDGFR, platelet-derived growth factor receptor; VEGFR, vascular endothelial growth factor receptor; FGFR, fibroblast growth factor receptor; CCK, colon carcinoma kinase; NGFR, nerve growth factor receptor; HGFR, hepatocyte growth factor receptor; EPHR, ephrin receptor; AXL, from the Greek word anex-elekto, or uncontrolled; TIE, tyrosine kinase receptor in endothelial cells; RYK, receptor related to tyrosine kinases; DDR, discoidin domain receptor; RET, rearranged during transfection; ROS, RTK expressed in some epithelial cell types; LTK, leukocyte tyrosine kinase; ROR, receptor orphan; MUSK, muscle-specific kinase; LMR, Lemur.
